# Comprehensive analysis of long non-coding RNAs in human breast cancer clinical subtypes

**DOI:** 10.18632/oncotarget.2454

**Published:** 2014-09-08

**Authors:** Xiaoping Su, Gabriel G Malouf, Yunxin Chen, Jianping Zhang, Hui Yao, Vicente Valero, John N Weinstein, Jean-Philippe Spano, Funda Meric-Bernstam, David Khayat, Francisco J Esteva

**Affiliations:** ^1^ Departments of Bioinformatics and Computational Biology, The University of Texas MD Anderson Cancer Center, Houston, TX, USA; ^2^ Groupe Hospitalier Pitié-Salpêtrière, Department of Medical Oncology, University Pierre and Marie Curie (Paris VI), Institut Universitaire de Cancérologie, AP-HP, Paris, France; ^3^ Breast Medical Oncology, The University of Texas MD Anderson Cancer Center, Houston, TX, USA; ^4^ Investigational Cancer Therapeutics, The University of Texas MD Anderson Cancer Center, Houston, TX, USA; ^5^ Breast Medical Oncology Program, New York University Cancer Institute, New York, NY, USA

**Keywords:** breast cancer, enhancers, expression profiling, lncRNA, RNA-Seq

## Abstract

Accumulating evidence highlights the potential role of long non-coding RNAs (lncRNAs) as biomarkers and therapeutic targets in solid tumors. However, the role of lncRNA expression in human breast cancer biology, prognosis and molecular classification remains unknown. Herein, we established the lncRNA profile of 658 infiltrating ductal carcinomas of the breast from The Cancer Genome Atlas project. We found lncRNA expression to correlate with the gene expression and chromatin landscape of human mammary epithelial cells (non-transformed) and the breast cancer cell line MCF-7. Unsupervised consensus clustering of lncRNA revealed four subgroups that displayed different prognoses. Gene set enrichment analysis for *cis*- and *trans*-acting lncRNAs showed enrichment for breast cancer signatures driven by master regulators of breast carcinogenesis. Interestingly, the lncRNA *HOTAIR* was significantly overexpressed in the HER2-enriched subgroup, while the lncRNA *HOTAIRM1* was significantly overexpressed in the basal-like subgroup. Estrogen receptor *(ESR1)* expression was associated with distinct lncRNA networks in lncRNA clusters III and IV. Importantly, almost two thirds of the lncRNAs were marked by enhancer chromatin modifications (i.e., H3K27ac), suggesting that expressed lncRNA in breast cancer drives carcinogenesis through increased activity of neighboring genes. In summary, our study depicts the first lncRNA subtype classification in breast cancer and provides the framework for future studies to assess the interplay between lncRNAs and the breast cancer epigenome.

## INTRODUCTION

Only 2% of RNAs encode for proteins in human cells. Although the large majority is not translated, RNAs play major roles in regulating transcriptional and non-transcriptional processes [1]. Long non-coding RNAs (lncRNAs) are eukaryotic RNAs longer than 200 nucleotides, with no coding capacity. Altered lncRNA expression has been associated with the development of cancer and other diseases [2]. Furthermore, several lncRNAs have shown promise as cancer biomarkers and potential therapeutic targets in several cancer subtypes [3-5]. However, the majority of those studies explored the role of a specific single lncRNA. Thus, comprehensive characterization of the landscape of lncRNAs in a cancer subtype has not been achieved because most genome-wide studies have used microarrays, which have the disadvantage of being biased toward the inclusion of probes that map the known protein-coding transcriptome [6].

Breast cancer is a heterogeneous disease with significant molecular variations, both between tumor subtypes and within a single tumor [7]. In 2000, Perou and colleagues proposed a molecular classification of breast cancer based on transcriptional profiling and cDNA microarrays [8]. Four main subtypes were identified and defined as basal-like, HER-2 enriched, luminal A, and luminal B [8, 9]. The PAM50 assay measures the mRNA expression levels of 50 genes and classifies breast cancers into the same subtypes [10]. Nevertheless, a classification of human breast cancer by lncRNA subtypes has not been established and the correlation between lncRNA subtype and mRNA expression has not been clarified [11]. Unfortunately, the panorama of lncRNAs in breast cancer has not been elucidated because this non-coding part of the genome was previously viewed as transcriptional noise [12]. In addition, genome-wide transcriptomic sequencing, which allows investigators to explore hundreds of tumors simultaneously, has only been available in recent years. Thanks to The Cancer Genome Atlas (TCGA) and the International Cancer Genome Consortium (ICGC) projects, we can conduct a comprehensive bioinformatic analysis to determine the panorama of lncRNAs across breast cancer subtypes.

The influence of lncRNAs is achieved by transcriptional interference, induced chromatin remodeling and histone modifications [13]. One of the best known lncRNAs, *HOTAIR*, affects the structure of chromatin through the polycomb repressive complex 2 (PRC2), and has been shown *in vivo* to promote breast cancer [14]. The discovery of *HOTAIR* as an independent prognostic factor in breast cancer was initially reported by Gupta and collaborators [14] and later validated in another cohort [15]. In addition, recent studies have shown that lncRNAs can be associated with enhancer regions, leading to increased activity of neighboring genes [16, 17]. Herein, we speculate that the identification of expressed lncRNAs, and more specifically those located in enhancer regions, may help to determine key lncRNAs involved in breast carcinogenesis. Targeting these key lncRNAs may then provide new therapeutic options for patients with breast cancer.

Using an important cohort that encompasses more than 600 samples from TCGA, we generated the first bioinformatic computation of the lncRNA subtype classification in a large cohort of breast cancer specimens that are fully clinically annotated. In addition, we performed an integrative analysis of the lncRNAs with mRNAs and chromatin histone modifications, with the aim of assessing the functional relevance of those lncRNAs. Our results unravel four subtypes of breast cancer with clinical relevance and provide the framework for future studies on lncRNAs in breast cancer.

## RESULTS

### Landscape of expressed lncRNAs in human breast cancer

**Figure 1:**
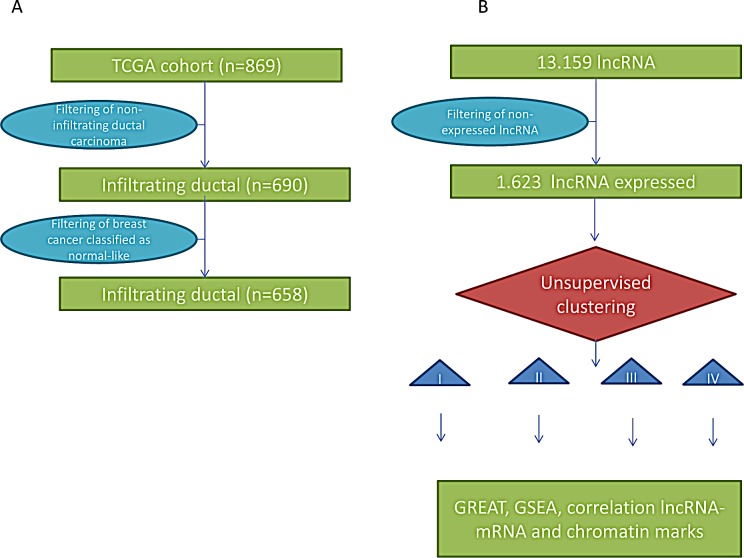
A) Flowchart for patient selection of breast cancer samples from The Cancer Genome Atlas (TCGA) project. B) Flowchart of methods used for analysis of lncRNAs.

While single lncRNAs have been previously shown to be specifically expressed in invasive breast cancers, the comprehensive catalogue of expressed lncRNAs remains unknown. To explore lncRNAs that play major roles in breast cancer, we reasoned that the FPKM (fragments per kilobase of non-overlapped exon per million fragments mapped) value of those lncRNAs must be greater than 1 in at least 10% of a large set of breast cancer samples. For the purpose of the analysis, we first extracted RNAseq data from TCGA, which included a total of 869 breast cancer samples. We selected 658 invasive ductal carcinomas that had transcriptomic classification data available (PAM50 assay). As a result, our cohort included 302 samples classified as luminal A, 167 samples classified as luminal B, 126 samples classified as basal-like, and 63 samples classified as HER2-enriched (Figure 1A; Table S1). We excluded samples that had normal-like breast cancer signatures because they may contain high proportions of contamination with normal tissue [10, 18]. We also excluded from this analysis other histologic subtypes of breast cancer (lobular, mucinous, etc.).

According to GENCODE gene annotation V15, which constitutes the largest manually curated catalogue of human lncRNAs, there are 13,159 lncRNAs that can be grouped into six categories based on their location with respect to protein-coding genes. There are 19,595 known coding genes in the human genome. The known lncRNAs include antisense RNAs (n=4424), large intergenic non-coding RNAs “lincRNAs” (n=6,421), sense overlapping transcripts (n=144), sense intronic transcripts (n=647), processed transcripts (n=1341) and 3-prime overlapping non-coding RNAs (n=37). After filtering the dataset to remove the lncRNAs that have low expression levels, we ended up with 1,623 expressed lncRNAs that are potentially relevant in breast cancer (Figure 1B; Table S2). Those lncRNAs include some already known to be involved in cancer, such as *H19* and *HOTAIR* (Table 1), as well as novel lncRNAs never reported in breast cancer (e.g., *HOTAIRM1*). The lncRNAs known to be expressed in other tumor types (e.g., prostate cancer) were not expressed in our dataset of 1,623 lncRNAs, which demonstrates tissue specificity (Table 1). We then asked whether the distributions of lncRNAs and mRNAs are different according to their FPKM values and find that the expression level of lncRNAs is very low as compared to the level of mRNAs (Figure S1), which is previously well known.

**Table 1:** Curated cancer-related lncRNAs extracted from the literature

lncRNA gene name	Expressed	Differentially expressed	Breast subtype classification	Functional annotation in the literature
*PCA3*	-	-	-	Prostate
*GAS5*	Yes	Yes	basal	Breast
*PVT1*	Yes	Yes	luminal B	breast, gastric
*DANCR*	Yes	Yes	basal	Breast
*PCAT1*	-	-	-	Prostate
*PCCEM1*	-	-	-	Prostate
*NEAT1*	Yes	Yes	luminal A	breast, ovarian
*KCNQ10T1*	-	-	-	colon, esophagus
*MALAT1*	Yes	Yes	luminal B	breast, colon lung, osteosarcoma
*HOTAIR*	Yes	Yes	HER2-enriched	Breast
*HOTAIRM1*	Yes	Yes	basal	-
*MEG3*	Yes	Yes	luminal A	brain, liver
*UCA1*	-	-		bladder cancer
*H19*	Yes	Yes	basal	bladder, breast, colon, kidney, liver, ovarian
*ANRIL*	-	-	-	Prostate
*XIST*	Yes	No	-	breast, colorectal, ovarian, testicular
*ZFAS1*	-	-	-	Prostate
*DLEU1*	-	-	-	Prostate
*RMST*	-	-	-	rhabdomyosarcoma
*HOST2*	-	-	-	Ovarian
*BIC*	-	-	-	B cell lymphoma
*NAMA*	-	-	-	Liver
*HULC*	-	-	-	papillary thyroid

To determine the biological relevance of the lncRNAs we identified, we applied a web-based analytic tool, GREAT, which analyzes the annotations of the neighboring genes [19]. We discovered that most lncRNAs were located in the vicinity of genes that played key roles in breast carcinogenesis (Table S2). Those include *ESR1, MAPT, GATA3, ZNF703, FOXA1, SOX4* and *SOX9*. Notably, we found that those lncRNAs were positively associated with the expression of the neighboring genes, suggesting that lncRNAs are *cis-*acting elements that influence master breast cancer regulators, and thus drive breast carcinogenesis (Table S2).

### Classification of lncRNA subtypes in human breast cancers

**Figure 2:**
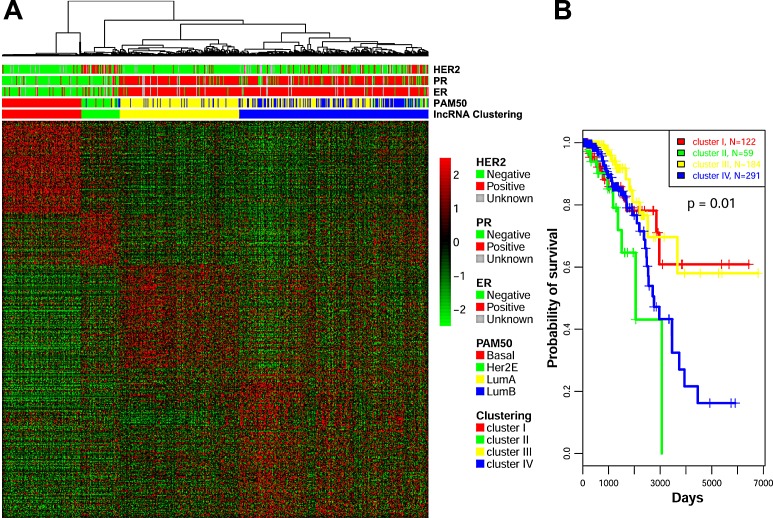
A) Unsupervised clustering of lncRNAs identified 4 clusters: cluster I (related to the basal-like breast cancer subtype), cluster II (related to the HER-2 enriched subtype), cluster III (related to luminal A subtype), and cluster IV (related to luminal A and B subtypes). Correlation with PAM50 classification, estrogen receptor (ER), progesterone receptor (PR) and HER2 status are depicted. B) Kaplan-Meier curve for overall survival in the 4 lncRNA transcriptomic classifications.

We then considered whether the lncRNAs expressed in breast cancer samples were associated with the transcriptomic classification based on PAM50. Our lncRNA-based unsupervised hierarchical consensus clustering revealed four subgroups (Figure 2A), which were highly correlated with the mRNA transcriptomic classification based on PAM50 (*p*=6.79×10^−243^) (Table S3). Interestingly, clusters I, II and III were highly correlated with the basal-like, HER2-enriched, and luminal A transcriptomic subtypes, respectively. Indeed, cluster III contained close to the majority of luminal A tumors (n=164; 89.13%). Conversely, cluster IV contained the majority of luminal B tumors (n=143; 85.6%), but also 46% (n=134/291) of luminal A tumors (Figure 2A; Table S3). Kaplan-Meier survival curves show that the four lncRNA groups display distinct lengths of overall survival (OS; *p*=0.01). Of note, the OS time corresponding to cluster III was better than that corresponding to cluster IV (Figure 2B). It is interesting that the median OS time for patients belonging to cluster III of the lncRNA-based classification was not reached, which was not the case for patients belonging to the luminal A subgroup as identified by PAM50 classification (Figure S2). However, the data were based on short follow-up periods; thus, it remains undetermined whether the lncRNA classification is better than the PAM50 classification for prognostic purposes. We conclude that there is cross-talk between lncRNA and mRNA. The principal component analysis we conducted showed similar patterns, confirming the robustness of our analysis (Figure S3). To further elucidate the clinical relevance of our lncRNA classifications, we investigated the molecular network governing each breast cancer cluster.

#### Cluster I – related to the basal-like breast cancer subtype (PAM50 classification)

**Figure 3:**
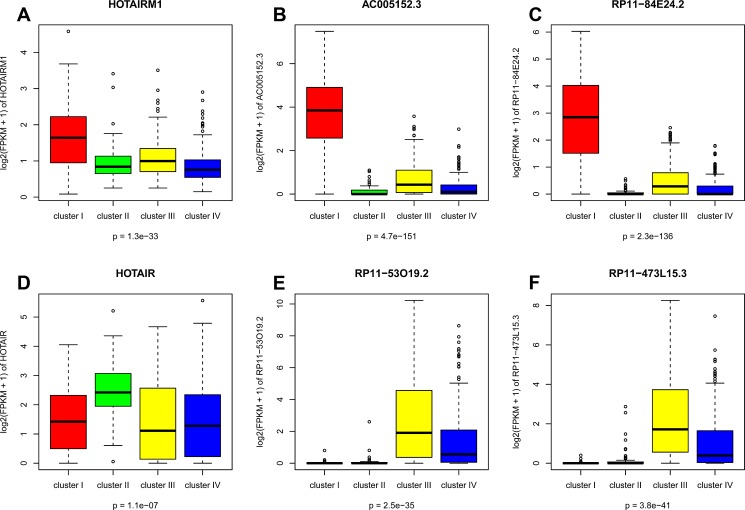
Boxplot for expression levels of lncRNAs: (A) *HOTAIRM1*, (B) *AC005152.3*, (C) *RP11-84E24.2*, (D) *HOTAIR*, (E) *RP11-53O19.2* and (F) *RP11-473L15.3*.

Overall, 122 lncRNAs were considered to be overexpressed in cluster I as compared to the other clusters, using a fold change (FC) ≥ 2 and a false discovery rate (FDR) < 0.05. The lncRNA *HOTAIRM1* was significantly overexpressed in this cluster. *HOTAIRM1* was previously shown to interact with polycomb repressive complexes 1 (PRC1) and 2 (PRC2), but was not reported to be involved in cancer (Figure 3A). Of note, *HOTAIRM1* expression was highly positively correlated with the expression of the *HOXA1* adjacent gene (Pearson correlation coefficient r=0.74) (Table S2). We also identified two lncRNAs, *AC005152.3* and *RP11-84E24.2*, with unknown functions among the top lncRNAs overexpressed in cluster I as compared to the other clusters (Figure 3B-C). Interestingly, the expressions of both of these lncRNAs were highly associated with the expression of the *SOX9* gene (r=0.43) (Table S2), and they were located within the vicinity of the *SOX9* gene, which was recently shown to determine the mammary stem cell state [20]. The list of all lncRNAs and their correlation with the mRNA expression of neighboring genes is provided in Table S2. The lncRNAs differentially expressed between the four subtypes are reported in Tables S4-S7, along with the corresponding correlations with copy number gains or losses.

#### Cluster II – related to the HER-2 enriched breast cancer subtype (PAM50 classification)

Overall, 57 lncRNAs were considered to be overexpressed in cluster II as compared to the other clusters. Importantly, the lncRNA *HOTAIR* located on chromosome 12q13.3 was significantly overexpressed in cluster II (FDR<0.0005; FC=2) (Figure 3D), and its expression was positively correlated with the expression of the adjacent gene *HOXC11* (r=0.84). The expression of *HOTAIR* was independent of the copy number gain.

#### Clusters III and IV – related to the luminal A and luminal B breast cancer subtypes (PAM50 classification)

Overall, 45 and 51 lncRNAs were identified as being overexpressed in clusters III and IV, respectively. The two top overexpressed lncRNAs (*RP11-53O19.2* and *RP11-473L15.3*) in cluster III were located within the vicinity of the *MRPS30* gene in the 5q12 chromosomal region, which has been associated with estrogen receptor (ER)-positive tumors as well as a favorable prognosis (Figure 3E-F) [21]. Moreover, these two lncRNAs were associated with positive expression of *MRPS30* (r=0.67).

### Identification of potential driver lncRNAs in breast cancer

The correlation between lncRNA expression and the expression of protein-coding genes has not been fully delineated. Whether lncRNAs are *cis*-acting (influencing neighboring genes) or *trans*-acting (influencing more distant genes) has not been determined [22-24]. Analysis of GENCODE v7 revealed that both *trans*- and *cis*-acting lncRNA expressions correlate with the expression of protein-coding genes [24]. Derrien et al. found more positive and extreme lncRNA-mRNA and mRNA-mRNA correlations in *cis-*acting elements than in *trans-*acting elements, and that the lncRNAs with expressions that significantly correlate with those of nearby protein-coding genes may be drivers [24]. To answer this question in breast cancer, we used GREAT to define the neighboring genes [19]. Using an FDR<0.05 and a fold change ≥ 2, we identified a set of overexpressed lncRNAs in each of the four lncRNA clusters, which included 122, 56, 45 and 51 lncRNAs in clusters I, II, III and IV, respectively. Of note, those lncRNAs were *cis*-acting on 203 protein-coding genes in cluster I, 96 protein-coding genes in cluster II, 96 protein-coding genes in cluster III, and 90 protein-coding genes in cluster IV. Furthermore, there was little overlap between those genes. Cluster I showed overexpression of the lncRNAs that influence their neighboring genes, *ALDH1A3* (a breast cancer stem cell marker), *SOX4, SOX9, and VIM*. Furthermore, we found those genes to be overexpressed in cluster I as compared to the other clusters: *ALDH1A3* (FC=2.7; FDR=2.9*10^−10^), *SOX4* (FC=1.6; FDR=1.7*10^−7^), *SOX9* (FC=2.7; FDR=5.8*10^−25^), and *VIM* (FC=1.8; FDR=1.3*10^−12^*)*. Cluster II (related to the HER2-enriched subtype) showed overexpression of the lncRNAs *cis*-acting on *HOXB2 and HOXC11* genes. Cluster III showed overexpression of the lncRNAs cis*-acting* on *GATA3, FOXA1* and *FOXD2*. Cluster IV showed overexpression of the lncRNAs *cis*-acting on *ZNF703, ESR1, WISP2* and *FGFR1* genes. Of note, *SOX4* is a master regulator of the epithelial-mesenchymal transition (EMT) in breast cancer [20], and is associated with overexpression of *LINC00340*, a *cis-*acting element in cluster I (basal-related). Importantly, the expression of the *SOX9* gene in cluster I was associated with the expression of the four *cis*-acting lncRNAs (Table S2).

Pathway analysis using GREAT showed that the lncRNAs that were overexpressed in cluster I are enriched for *cis*-acting genes that belong to the following 4 gene sets: (1) up-regulated genes in the basal-like subtype of breast cancer (*p*=6.78*10^−7^); (2) 100 transcription regulators showing the most correlated expression with the 9 “embryonic stem cell” transcription factors that are preferentially and coordinately overexpressed in high-grade, ER-negative breast cancer (*p*=1.93*10^−8^); (3) genes down-regulated in bone relapse of breast cancer (*p*=5.47*10^−8^) and (4) down-regulated genes from the optimal set of 550 markers discriminating breast cancer samples by *ESR1* [Gene ID=2099] expression: ER(+) vs ER(−) tumors (*p*=7.48*10^−5^).

Puzzlingly, we did not find any pathway enrichment for cluster II, which raised questions about the role of lncRNAs in HER2-enriched tumors. Cluster III exhibited enrichment for genes that are down-regulated in basal-like breast cancer (*p*=3.32*10^−8^) and for genes that are down-regulated in brain relapse of breast cancer (*p*=1.43*10^−5^). Cluster IV was enriched for genes that are up-regulated in the luminal B subtype of breast cancer (*p*=3.9*10^−6^) and for genes that are down-regulated in breast cancer tumors (formed by MCF-7 xenografts) that are resistant to tamoxifen [PubChem=5376] (*p*=2.58*10^−8^). These results are remarkable because the luminal B subtype has been associated with resistance to hormonal treatment even in the presence of estrogen and/or progesterone receptor overexpression [25]. We then looked at the lncRNAs near *ESR1*, *GATA3*, and *FOXA1* and analyzed if their expression is only coordinated in luminal breast cancer but not in basal-like breast cancer as the high expression of these genes is characteristic in luminal breast cancer. As expected, the expression levels of 3 of those lncRNAs were significantly highly expressed in luminal subtypes as compared to basal subtypes (Figure S4).

Thus, we conclude that it is likely that genes located in the vicinity of those lncRNAs are co-regulated and play an important role in driving tumor resistance to endocrine therapy. Additional studies will be needed to determine whether tumors classified to lncRNA cluster IV represent the subgroup of patients with hormone-receptor–positive breast cancer that is resistant to hormonal therapy.

### A subset of lncRNAs associated with enhancers

**Figure 4:**
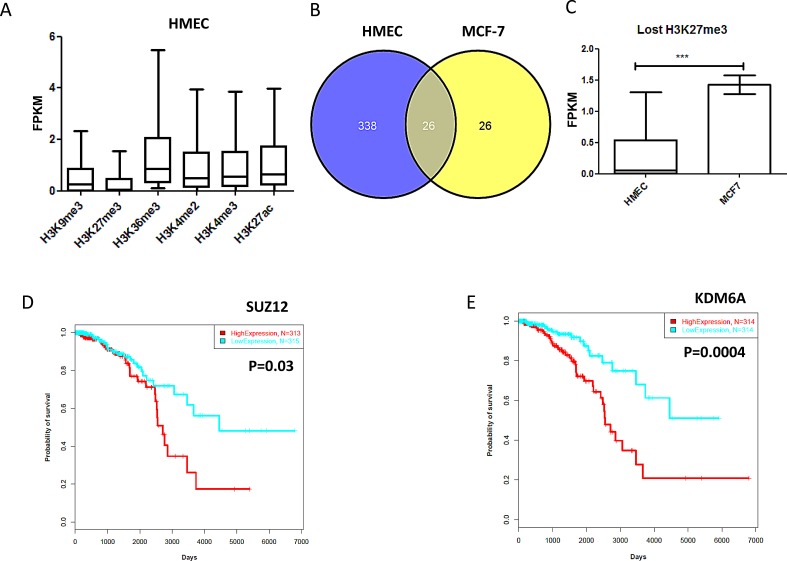
A) Boxplot for gene expression using FPKM for lncRNAs according to corresponding histone marks. Note that the lncRNAs marked by inactive histone marks H3K27me3 and H3K9me3 have low expression. B) Venn diagram for the number of lncRNAs marked by H3K27me3 in HMECs and MCF-7. C) Bar graph for median lncRNA expression level of genes with H3K27me3 mark in HMECs, as compared to genes without this mark in MCF-7 cells. Note that lncRNAs that lost H3K27me3 had increased gene expression. D) Kaplan-Meier curves for overall survival time in patients with breast cancer according to *SUZ12* expression. E) Kaplan Meier curves for overall survival time in patients with breast cancer according to *KDM6A* expression.

Previous studies have reported that the patterns of lncRNA expression show specificity to the cell type and are likely to be controlled epigenetically [26]. Furthermore, recent studies have revealed that lncRNAs display enhancer-like functions.^17^ Thus, we analyzed the relationship between the 1,623 lncRNAs we identified in TCGA breast cancer data and different histone markers. As expected, H3K27me3 and H3K9me3 were associated with repressed genes, and H3K4me3, H3K4me2, H3K36me3, and H3K27ac were associated with expressed genes in human mammary epithelial cells (HMECs) (Figure 4A). Similar findings were observed for the breast cancer cell line MCF-7 (not shown). Of note, 197 lncRNAs are bivalent (H3K4me3/H3K27me3) in HMECs, as compared to 34 lncRNAs in MCF-7 cells. Out of those, we can mention *HOTAIRM1*, which was expressed in HMECs and marked by H3K4me3, but was bivalent and repressed in MCF-7 cells. Strikingly, the majority of lncRNAs marked by H3K27me3 (a polycomb mark) in HMECs were not identified in MCF-7 cells (Figure 4B; Table S8), which is consistent with their gene expression changes (Figure 4C). These data highly suggest a putative role of H3K27me3 demethylase *UTX (KDM6A)* in the derepression of these lncRNAs in breast cancer cells, through a mechanism identical to that of coding genes. We then considered whether the H3K27me3 demethylase *UTX* or the polycomb complex genes (*EED, SUZ12*, and *EZH2*) were associated with the patient’s outcome. Strikingly, only *SUZ12 and KDM6A* were associated with a poor outcome; *EZH2* was not (Figure 4D-E).

We then investigated whether lncRNAs are associated with enhancers, which are defined as genomic regions marked by acetylation with H3K27ac. Strikingly, 1,038 out of 1,623 were found to be marked by H3K27ac in HMECs and/or MCF-7 cells, suggesting that those lncRNAs may act as enhancers. Using more stringent criteria by defining the enhancers as regions bearing H3K27ac and H3K4me2, we identified 937 lncRNAs in HMECs that had both marks.

As expected, gene set enrichment analysis (GSEA) showed that *HOTAIR*, which is overexpressed in cluster II, was associated with genes with high-CpG-density promoters (HCPs) that are marked with H3K27me3 in precursor cells of brain and neural tissue [27]. Unexpectedly, we discovered a novel association of differentially expressed lncRNAs with histone marks. For instance, *H19*, which is overexpressed in the basal-like breast cancer subgroup, was enriched for genes with HCP marked by H3K4me2 and H3K4me3 [27], suggesting that *H19* may interact with the trithorax group of proteins, which maintains gene expression. Strikingly, the overexpression of *MEG3* and *RP11-417E7.2* was associated with the enrichment of bivalent genes in different samples, including embryonic stem cells. Although these data are important, further validation is needed to clarify the role of those lncRNAs in breast cancer.

### Integrative analysis of lncRNA-mRNA functional associations

The expression of lncRNAs in loci known to function transcriptionally has been shown to correlate with the expression of the coding transcripts at those loci [28]. However, according to the category to which the lncRNAs belong (*cis-*antisense, intronic, or bidirectional), the correlation with their associated protein-coding genes may differ. In fact, the expression of both intronic and bidirectional lncRNAs has been shown to correlate with the expression of their associated coding genes [28, 29]. For example, we identified the lncRNA *RP3-443C4.2* within the vicinity of estrogen receptor 1 (*ESR1*), a gene with important functions in breast cancer. The expression profiles of *ESR1* and the lncRNA *RP3-443C4.2* were highly correlated. However, *RP3-443C4.2* was significantly overexpressed in cluster IV, but not in cluster III, suggesting a distinct regulation of *ESR1* in cluster III versus that in cluster IV. Of note, *RP3-443C4.2* was positively correlated with the expression of 24 neighboring and distant genes, including *ESR1, GATA3*, and *ZNF703*. *ZNF703* is an oncogene commonly associated with luminal B breast cancer, and was previously shown to have differential control of luminal and basal progenitors in epithelial cells of the breast [30].

Consistent with a previous report, we found general correlation between the expression of *cis-*antisense lncRNAs and pairs of protein-coding genes [29]. For example, a *cis-*antisense lncRNA, *MAPT-AS1*, exhibits a positive correlation (r=0.70, *p*<10^−16^) with its sense protein-coding gene, *MAPT*. This gene has an essential role in determining the breast tumor response to paclitaxel [31]. Moreover, *MAPT-AS1* was highly correlated with the expression of the progesterone receptor (*PGR* and *TMEM26*). Strikingly, this lncRNA was not expressed at all in clusters I and II of our lncRNA classification of breast cancer (Figure S5).

### Association of lncRNAs with overall survival time

**Figure 5:**
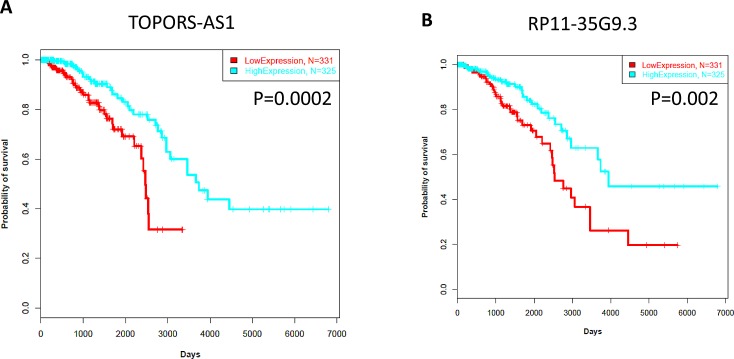
A) Kaplan-Meier curves for overall survival time in patients with breast cancer according to expression of *TOPORSAS1*. B) Kaplan-Meier curves for overall survival time in patients with breast cancer according to expression of *RP11-35G9.3*.

We considered whether lncRNA expression is associated with patient outcome. We used a Cox model in which all the 1,623 lncRNAs were correlated with overall survival in the TCGA cohort. We found 6 lncRNAs to be associated with patient outcome (FDR<0.05). Two of them (*TOPORS-AS1, RP11-35G9.3*) were associated with a good outcome (Figure 5A-B). Interestingly, these lncRNAs were associated with a loss of the activating mark H3K36me3 in breast cancer cell line MCF-7 as compared to that in human mammary epithelial cells (HMECs). This is consistent with their repression in cancer and suggests that they act as tumor suppressors. Of note, topoisomerase I binding, arginine/serine-rich, E3 ubiquitin protein ligase (*TOPORS*) is considered to be a probable tumor suppressor that is involved in cell proliferation and apoptosis through the regulation of p53/TP53 stability via ubiquitin-dependent degradation.

## DISCUSSION

To the best of our knowledge, this study represents the first comprehensive description of expressed lncRNAs in breast cancer, as identified through bioinformatic analysis of RNAseq data in a large patient cohort that encompasses 658 infiltrating ductal carcinomas of the breast. Our in-depth genomic analyses unraveled several novel findings. First, we identified the expression of 1,623 lncRNAs that are likely to play important roles in breast cancer initiation and progression, and connected their expression with chromatin marks of HMECs and the MCF-7 breast adenocarcinoma cell line. As expected, our bioinformatic approach validated previous observations such as the link between *HOTAIR* expression and the PRC2 complex in breast cancer [14], giving us confidence in our methodology. We identified several novel lncRNAs that had not been previously reported in breast cancer. For example, we found that the *HOTAIRM1* was overexpressed in the basal-like subtype of breast cancer. Of note, the expression of *HOTAIRM1* was previously shown to be specific to the myeloid lineage of hematopoietic cells [32]. Mechanistically, *HOTAIRM1* may act by modulating gene expression in the *HoxA* gene cluster. Thus, further studies are needed to clarify its role in the basal-like subtype of breast cancer.

Second, we provided a new molecular classification of breast cancer using lncRNA expression. Indeed, according to this classification, we found that samples related to lncRNA clusters I and II highly overlap with the basal-like and HER2-enriched subgroups, respectively. Conversely, lncRNA clusters III and IV display a completely different distribution of luminal A and luminal B samples. These data are important because the unsupervised clustering of breast cancers was independent from the *ER*, HER2 and PR status. This classification may outperform the PAM50 classification for predicting a patient’s response to hormonal therapy and prognosis. However, in the absence of important information on clinical follow-up and clinical response to hormonal therapy, such comparisons cannot be made and will require future studies.

Another important topic is the interplay between lncRNA and chromatin marks. We discovered that almost two thirds of the lncRNAs expressed in breast cancer are localized at enhancer regions. Identifying and targeting those enhancers may provide new therapeutic opportunities for breast cancer. More importantly, the majority of lncRNAs marked by H3K27me3 in normal breast tissue (HEMC) did not show that mark in the MCF-7 cancer cell line. We speculate that H3K27me3 demethylase may play a role in this process. Of note, *UTX* overexpression was associated with poor patient outcome in our series, which was not the case for *EZH2*. This finding is in accordance with a recent report showing that *UTX* overexpression is associated with poor outcome in breast cancer.^33^ In contrast with previous studies, however, we did not observe a negative impact of *EZH2* expression on patient outcome [34].

Our study identified two lncRNAs, *TOPORS-AS1* and *RP11-35G9.3*, that may act as tumor suppressors because their overexpression was associated with a good outcome. Notably, these 2 lncRNAs were marked by H3K36me3 in HMEC, but not in the MCF-7 breast cancer cell line, which is consistent with their repression. Mechanistic studies are needed to clarify the role of those lncRNAs on tumor proliferation and invasion. If validated in an independent cohort, those lncRNAs may serve as robust biomarkers.

Future studies should also focus on the role of lncRNAs in shaping chromatin. Our integrative analysis of chromatin modifications with lncRNAs identified several lncRNAs that were associated with histone marks (e.g., H3K4me3 and H3K4me2 for *H19*) in normal breast tissue and breast cancer cell lines. *HOTAIR* and *PCAT-1* were previously shown to interact with the PRC2 complex [14, 35].

From a clinical standpoint, our preliminary data indicate that lncRNA-based clustering identifies variations in patient prognosis; however, we cannot make definitive conclusions because outcomes data are very limited in the TCGA database. Additional studies will be needed to compare the new lncRNA-based classification of breast cancer with PAM50 and other classifications based on transcriptomics. From a therapeutic perspective, *MAPT-AS1*, the antisense lncRNA for *MAPT* (tau protein), was previously shown to exhibit sensitivity to paclitaxel [31]. We identified *MAPT-AS1* to be exclusively expressed in clusters III and IV (compared to clusters I and II), which is consistent with luminal A and B breast cancers. We found a positive correlation between *MAPT-AS* and *MAPT*. However, *MAPT-AS* may also serve as a predictive and prognostic marker in breast cancer, as previously demonstrated [36].

Our study represents the first comprehensive analysis of lncRNAs in breast cancer, with integrative analysis revealing that the majority of those lncRNAs act as enhancers. These data provide a rationale for targeting lncRNAs in breast cancer, and suggest that lncRNAs may be used in the future to predict response to treatment as well as patient outcome. We believe this study sets the stage for a new framework for future research in the role of lncRNAs in breast cancer.

## MATERIALS AND METHODS

### The Cancer Genome Atlas (TCGA) Data

TCGA breast cancer RNA-Seq data (bam files) and their related clinical data were obtained from the Cancer Genomics Hub (CGHub, https://cghub.ucsc.edu/) and TCGA Data Portal (https://tcga-data.nci.nih.gov/tcga/). The paired-end FASTQ files for each sample were extracted from bam files using bam2fastq (http://www.hudsonalpha.org/gsl/information/software/bam2fastq).

### ChIP-Seq data

ChIP-Seq peak data for histone marks H3K4me3, H3K4me2, H3K36me3, H3K27ac, H3K27me3 and H3K9me3 in both human mammary epithelial cells (HMECs) and breast cancer cell line MCF-7 were obtained from the UCSC ENCODE Histone Modification Tracks (http://genome.ucsc.edu/cgi-bin/hgTrackUi?hgsid=331813161&c=chr21&g=wgEncodeHistoneSuper). To examine the histone modification profiles of lncRNA genes, we analyzed the promoter regions of *lncRNA* genes for overlap with histone mark enrichment peaks. Specifically, the lncRNA was defined to be marked/associated with a specific histone mark if the peak from ChIP-Seq data for a specific histone mark was located within +/− 5kb from the transcription start site (around the promoter regions) for the lncRNA.

### Mapping/Alignment

The raw, paired-end reads in FASTQ format were then aligned to the human reference genome, GRCh37/hg19, using MOSAIK alignment software [37]. MOSAIK works with paired-end reads from Illumina HiSeq 2000, and uses both a hashing scheme and the Smith-Waterman algorithm to produce gapped optimal alignments and to map exon junction-spanning reads with a local alignment option for RNA-seq. The resulting alignments were then saved as a standard bam file.

### The raw counts for each gene of both mRNAs and lncRNAs from RNA-seq

We then counted the mapped reads in genomic features such as genes (mRNAs and lncRNAs) annotated in GENCODE15 to generate the raw counts for each gene using the HTSeq-count script distributed with the HTSeq package. We chose the “union” mode of HTSeq to mask the regions that overlapped between mRNAs and lncRNAs to overcome the issue of non-strand-specific RNA sequencing in the kit (TruSeq) used in TCGA data.

### Count data normalization

Raw reads count data were normalized across samples with DESeq_1.10.1 [38]. Specifically, DESeq first estimates the effective library size, which is also called size factor, by dividing each column by the geometric means of the rows given a matrix or data frame of raw count data. Then, the median of these ratios (skipping the genes with a geometric mean of zero) are used as the size factor for that column. With the estimation of size factors, DESeq then divides each column of the count table by the size factor for that column. By doing that, the count values are brought to a common scale, making them comparable across samples. Furthermore, we transformed the count data by the varianceStabilizingTransformation function provided in the DESeq package. With this function, the standard deviation of each gene is roughly constant regardless of the gene expression magnitude.

### FPKM calculation

We calculated the number of fragments per kilobase of non-overlapped exon per million fragments mapped (FPKM). Since the raw count data per gene were generated with the “union” mode in HTSeq, where the reads mapped to the overlapping regions between mRNAs and lncRNAs were not counted, the exon sequences for which overlap between mRNAs and lncRNAs exists were excluded when we calculated the gene lengths for both mRNAs and lncRNAs.

### Low expression filtering

To reduce noise, we kept only mRNAs or lncRNAs with FPKM equal to or above 1 in at least 10% of the samples for downstream analysis.

### Detection of differential mRNA and lncRNA expressions

All statistical analyses were performed using the R and R-Bioconductor statistical programming environment. We identified differentially expressed mRNAs and lncRNAs using DESeq with the standard comparison mode between the two experimental conditions. *P* values were adjusted for multiple testing with an embedded Benjamini-Hochberg procedure in DESeq.

### Consensus clustering by lncRNAs

To assess the stability of the discovered clusters, we performed consensus clustering. We conducted 500 runs of hierarchical clustering on the resampled data. For each run, 80% samples and 80% lncRNAs were randomly chosen. The distance measurement was set as a Pearson correlation, and the linkage function was set as “Ward.” Based on the 500 runs, a consensus was obtained by taking the average over the connectivity matrices of every perturbed dataset. Then we carried out hierarchical clustering with the consensus matrix as a similarity matrix, with “Euclidean” as the distance measurement and “Ward” as the linkage function. We also calculated the Bayesian information criterion to detect the number of clusters.

### Correlation matrix of lncRNA-mRNA

We generated a correlation matrix between lncRNAs and mRNAs by computing the Pearson correlation coefficient between all pairs of significant lncRNAs and mRNAs. A matrix was constructed with entries in the ternary scale (−1, 0, 1), where the top 1% with negative correlation was assigned -1; the top 1% with positive correlation was assigned 1; and the others were assigned 0. The matrix was clustered and visualized using a Euclidian distance metric and complete linkage clustering.

### Gene set enrichment analysis (GSEA)

In order to associate functional gene sets to each *lncRNA*, we performed GSEA as previously described [39, 40]. Specifically, we used each lncRNA as a profile/phenotype, and computed the Pearson correlation coefficient for each lncRNA-mRNA combination. For each lncRNA, mRNAs were ranked according to the Pearson correlation coefficient to generate ranked gene (mRNA) lists for GSEA using 10,295 functional gene set collections from the GSEA Molecular Signatures Database. Gene sets with a false discovery rate (FDR) below 5% were considered significant, and the GSEA normalized enrichment scores (NES) were transformed to a ternary scale (−1, 0, 1), where FDR>0.05 was assigned a value of 0; FDR<0.05 & NES>0 was assigned 1; and FDR<0.05 & NES<0 was assigned -1. We used R package GSA^41^ to perform GSEA and to construct an association matrix of each lncRNA, with each of 10,295 functional gene sets whose entries are the ternary scale (−1, 0, 1) prior to hierarchical clustering. We then performed biclustering on this matrix to identify significant lncRNAs associated with functional gene sets.

### Disclosure of potential conflicts of interest

The authors declare no conflicts of interest.

## SUPPLEMENTARY FIGURES AND TABLES



















## Abbreviations

EMT: epithelial-mesenchymal transition
ER: estrogen receptor
FC: fold change
FDR: false discovery rate
FPKM: fragments per kilobase of non-overlapped exon per million fragments mapped
GSEA: gene set enrichment analysis
HCP: high-CpG-density promoter
HER2: human epidermal growth factor receptor &#x2013# 2
HMEC: human mammary epithelial cell
ICGC: International Cancer Genome Consortium
lncRNA: long non-coding RNA
NES: normalized enrichment score
OS: overall survival
PR: progesterone receptor
PRC1, PRC2: polycomb repressive complex 1, polycomb repressive complex 2
r: Pearson correlation coefficient
TCGA: The Cancer Genome Atlas
TOPORS: topoisomerase I binding, arginine/serine-rich, E3 ubiquitin protein ligase
