# Supplementary material for: Comprehensive analysis of long non-coding RNAs in human breast cancer clinical subtypes
Source: Oncotarget. 2014 Sep 8;5(20):9864–76. doi: 10.18632/oncotarget.2454 (PMC4259443; doi:10.18632/oncotarget.2454)
Supplement: Supplementary file 1 [file oncotarget-05-9864-s001.pdf]

Comprehensive analysis of long non-coding RNAs in human breast cancer clinical subtypes

Supplementary Material

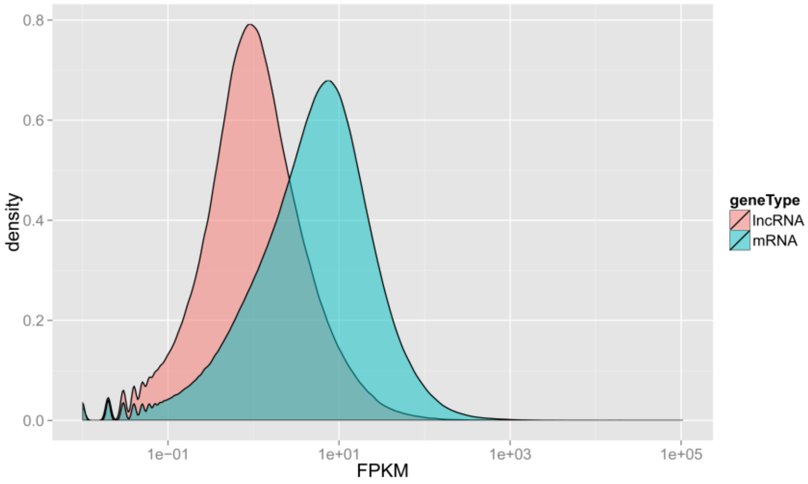

Supplemental Figure S1: FPKM density plots of mRNAs and lncRNAs expression in the breast dataset of The Cancer Genome Atlas (TCGA)

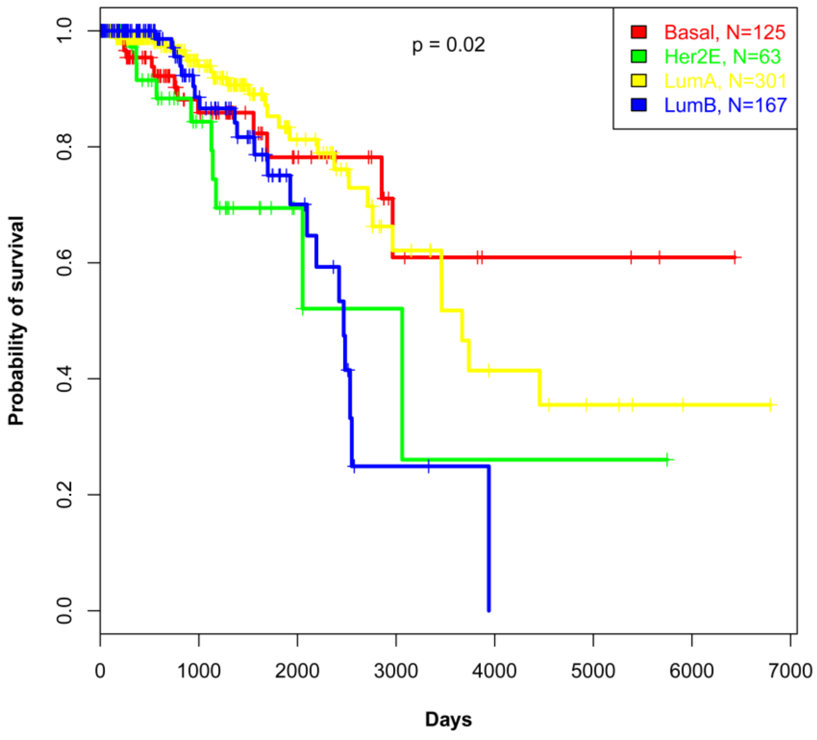

Supplemental Figure S2: Kaplan-Meier curves for overall survival time in patients with breast cancer according to PAM50 classification.

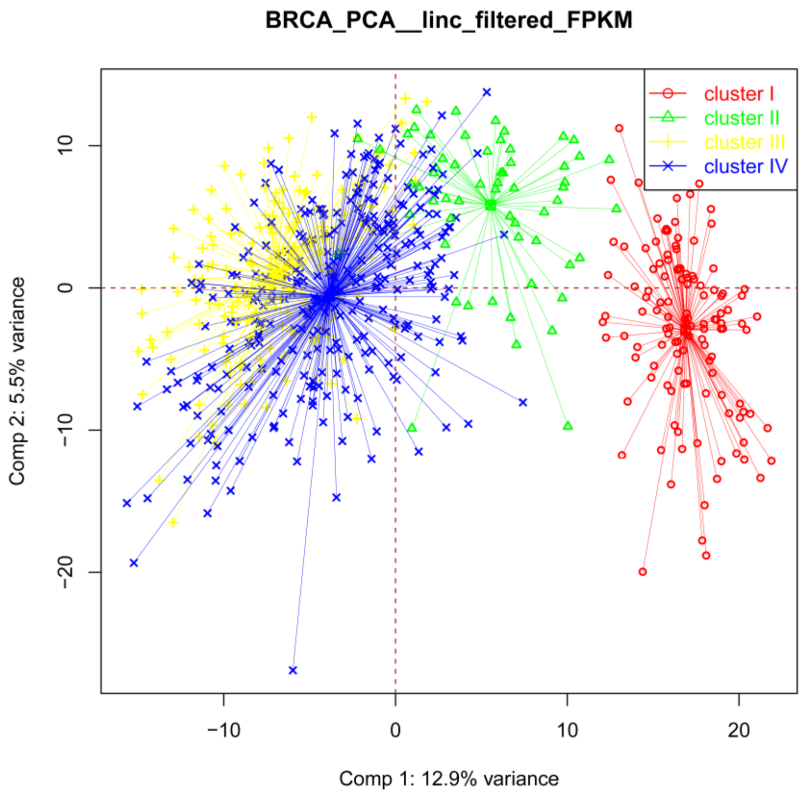

Supplemental Figure S3: Principal component analysis of lncRNA datasets. Results were projected on the first 3 components, and show 4 main sample clusters highlighted in different colors. BIC is Bayesian information criterion.

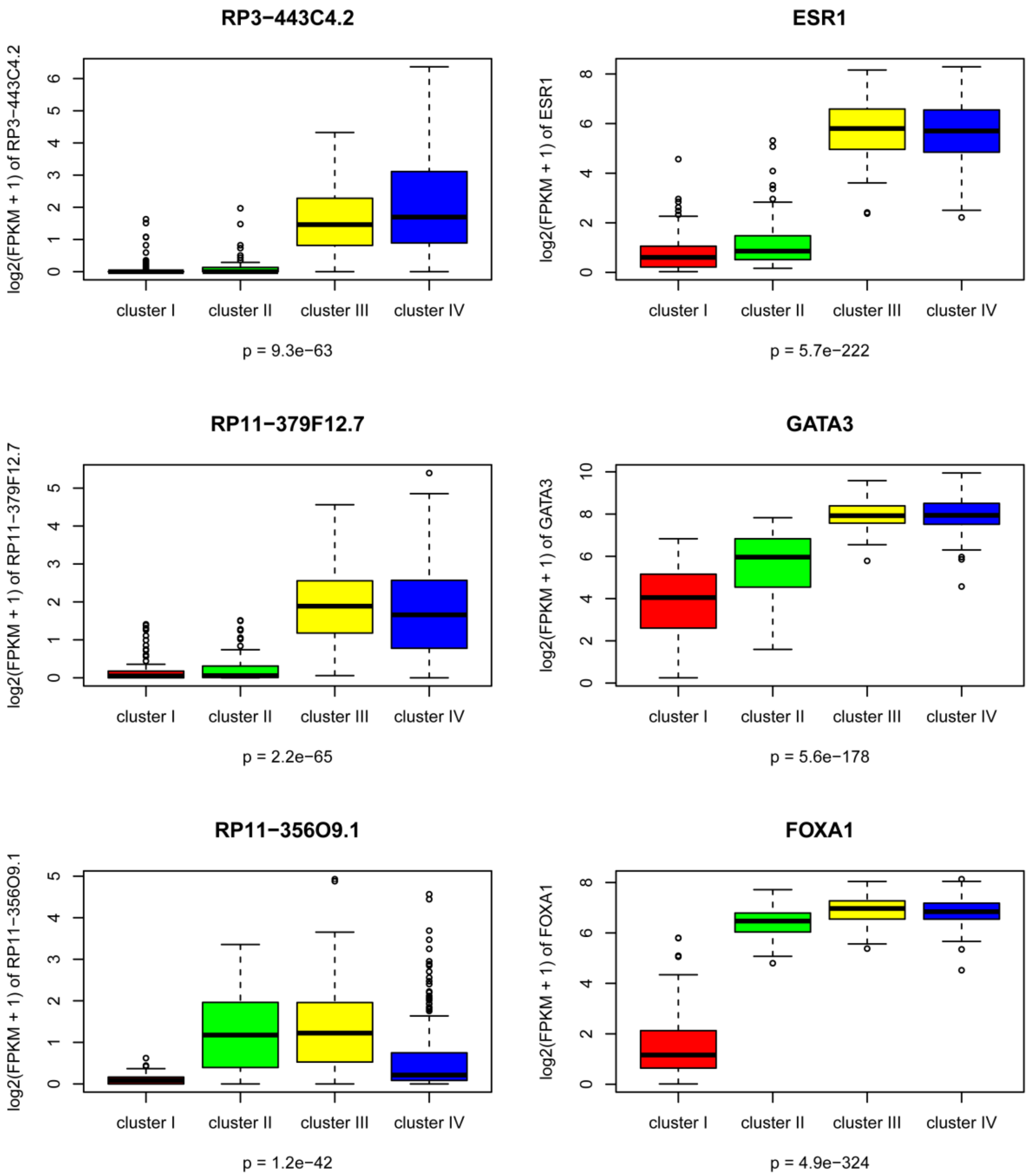

Supplemental Figure S4: Box-plots for the expression levels of *ESR1*, *GATA3* and *FOXA1* as well as their neighboring lncRNAs showing their coordinated expression in luminal breast cancer but not in basal-like breast cancer as the high expression of these genes is characteristic in luminal breast cancer.

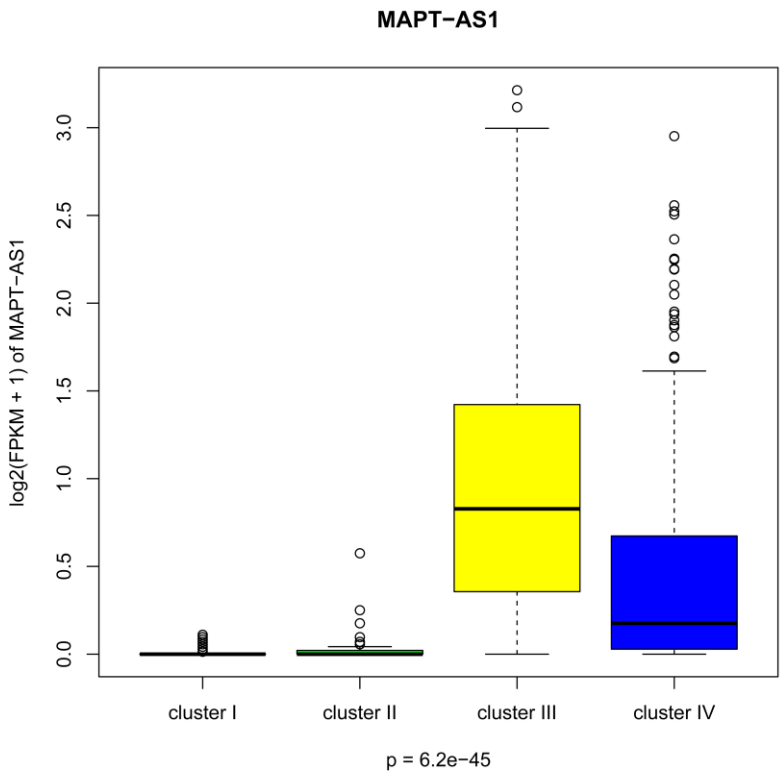

Supplemental Figure S5: Boxplot for expression levels of MAPT-AS in the 4 subgroups of lncRNA classification.
